# Determination of the consequences of VHL mutations on VHL transcripts in renal cell carcinoma

**DOI:** 10.3892/ijo.2012.1561

**Published:** 2012-07-20

**Authors:** CLAIRE TAYLOR, RACHEL A. CRAVEN, PATRICIA HARNDEN, PETER J. SELBY, ROSAMONDE E. BANKS

**Affiliations:** 1 Cancer Research UK Cancer Centre Genomics Facility, Leeds Institute of Molecular Medicine;; 2 Clinical and Biomedical Proteomics Group, Leeds Institute of Molecular Medicine;; 3 Department of Histopathology, St. James’s University Hospital, Leeds, UK

**Keywords:** von Hippel-Lindau, mutation screening, mRNA, transcript, renal cancer

## Abstract

Genetic and epigenetic changes in the von Hippel-Lindau (VHL) tumour suppressor gene are common in sporadic conventional (clear cell) renal cell carcinoma (ccRCC). The effects on VHL expression are unknown but increased understanding may be relevant clinically, either in terms of prognosis or in therapy selection. We have examined the expression of *VHL* mutant RNA in 84 ccRCC tumours previously screened for mutations in genomic DNA, 56 of which contained 52 unique mutations or polymorphisms. Based on the predicted change to the primary amino acid sequence, 24 of the mutations were missense, 11 resulted in frameshifts with premature truncation, 9 resulted in immediate truncation at the site of the mutation and 2 were frameshifts which extended the reading frame beyond the normal stop codon. Nine tumours had intronic variants, including substitution of invariant residues at splice sites, deletion of nucleotides spanning the exon-intron junction, an intronic variant of unknown function and the polymorphism c.463+43A>G. Four variants were identified which were present in genomic DNA but not in mRNA. Three of these, all encoding apparent missense changes to the primary amino acid sequence, were located close to the ends of exons, reduced the strength of the splice site and function as null rather than missense variants. One nonsense variant was not detectable in mRNA but all other mutations resulting in premature truncation codons (PTCs) were, suggesting truncating *VHL* mutations may potentially generate truncated VHL protein. An intronic variant, c.341-11T>A, previously regarded as of unknown function, is associated with an increased level of skipping of exon 2 and may, therefore, reduce production of pVHL. Our data show that the biological consequences of *VHL* mutations are not necessarily predictable from the sequence change of the mutation and that for the majority of VHL mutations, the potential for the generation of mutant protein exists.

## Introduction

Over 200,000 cases of renal cancer are diagnosed annually, with ∼75% of renal cell carcinomas being of the clear cell subtype (ccRCC). Loss of function of the von Hippel-Lindau (VHL) tumour suppressor gene occurs in both sporadic ccRCC and VHL disease, a familial cancer syndrome predisposing to a variety of malignant and benign tumours including RCC (1,2). The germline mutation spectrum in patients with VHL disease includes exon deletion, as well as nonsense, frameshift, splice site and missense mutations (3). *VHL* is a classical tumour suppressor gene and somatic inactivation of the second copy by LOH, methylation or mutation is observed in tumours from VHL patients (2,4). The *VHL* gene has also been implicated in the majority of sporadic ccRCC cases, with mutation frequencies of 75–82% reported (5,6). Together with loss of heterozygosity (LOH) and promoter methylation, the evidence implicates biallelic inactivation of *VHL* in 86% of ccRCC (6).

The *VHL* gene on chromosome 3p25, consists of three exons (1,7). It produces two transcripts, and is expressed in a wide range of tissues and developmental stages (1,7). The larger transcript contains all three exons and encodes two biologically active protein isoforms, pVHL19 and pVHL30. The longer isoform contains all 213 residues of the *VHL* ORF whereas the shorter isoform uses an internal translation start site (8). The shorter, less abundant transcript (Δ2) lacks exon 2 (2,9). No protein product from Δ2 has been identified, although in-frame. A non-expressed processed pseudogene is present on chromosome 1q12 (10).

The primary amino acid sequence of *VHL* has little similarity to other sequences in the human genome (1,11) but conservation amongst mammals is high. The main exception is the N-terminus which in humans and other higher primates contains eight copies of an acidic pentamer repeat but has fewer copies in rats and mice. Lack of mutation coupled with the relatively low evolutionary conservation of this repeat motif and its absence from the pVHL19 isoform suggests it is of lesser importance to pVHL function than the rest of the gene (11).

The role played by pVHL as the substrate recognition domain of an E3 ubiquitin ligase complex targetting HIF-α for ubiquitination and degradation by the proteasome is well understood and stabilisation of HIF-α (in particular HIF-2α) resulting from loss of *VHL* function in RCC has been shown to be central to tumourigenesis (12). However, the relative importance of HIF-independent pVHL activity remains to be determined. Knowledge relating to the consequences of disruption of VHL with stabilisation of HIF and consequent upregulation of factors such as vascular endothelial growth factor (VEGF) has been exploited with drugs targeting these pathways such as sorafenib, sunitinib and bevacizumab which have improved response rates and relapse-free survival in RCC (13).

Truncating and missense mutations are rarely reported in the first half of exon 1 but are otherwise distributed across the entire coding sequence (3,6). Any correlations between mutation type and aspects of phenotype as observed in familial VHL (3) have not yet been found in sporadic RCC although such information might be of value prognostically and in treatment response. Following our previous analysis of the genetic and epigenetic status of *VHL* in tumour specimens from a large series of patients with sporadic RCC (6,14), we have extended the characterisation of a subset of these samples to examine the functional consequences of such changes on the *VHL* transcript to understand further the potential biological impact.

## Materials and methods

### Samples and RNA and DNA extraction

Frozen tissue samples from 84 patients with RCC (80 conventional and 4 other subtypes), previously screened for VHL mutation, methylation and LOH and covering a spectrum of changes (6,14) were selected for analysis together with 11 RCC cell lines (Table IA–C). For tissue samples, 5–10 sections were cut for RNA extraction from frozen samples embedded in OCT, with flanking sections used to determine the percentage of tumour cells. Sections were immersed in 500 μl of RNAlater (Qiagen: http://www1.qiagen.com) and stored at −20°C for 24–72 h. Total-RNA was extracted using RNeasy (Qiagen) following the manufacturer’s animal tissue protocol and assessed using a Nanodrop spectrophotometer. RNA was extracted from cultured cells using RNeasy, and DNA using QIAamp (Qiagen), following the animal cell protocol.

### Mutation screening of cell line DNA

Screening for mutations in genomic DNA from cell lines was carried out by direct DNA sequencing using the primers and conditions previously described (6).

### RT-PCR and PCR

*VHL* mRNA was amplified using primer set 9 (codons 1–170) and/or primer set 3 (codons 89 to 214) depending on the location of the mutation. Primer sequences are: set 3 forward (M3537) cgtcgtgctgcccgtatg and reverse ccatcaaaagctgagatgaaacag (M3548); set 9 forward cccgggtggtctggatcg (M3529) and reverse tggcaaaaataggctgtcc (M3540). RT-PCR was specific for fully processed *VHL* mRNA and did not amplify unprocessed *VHL* pre-mRNA, *VHL* from co-purified genomic DNA or the processed *VHL* pseudogene. To achieve this, the 3′ end at least one of each pair of primers was positioned over a nucleotide which discriminates between *VHL* and the pseudogene and each primer pair spanned at least one exon-exon junction. RNA-specificity of RT-PCT products was further verified by showing the absolute RT-dependence of the reaction by heat-inactivation of the reverse transcriptase prior to addition of RNA (data not shown). Sequencing of RT-PCR products confirmed that they were derived from the gene and not from the pseudogene. The *VHL* splice variant lacking exon 2 (Δ) co-amplified with the full length product when using primer set 3.

One step RT-PCR was carried out using Superscript III one-step RT-PCR kit (Invitrogen). Reactions contained 10 ng of total RNA, 10 pmol forward primer, 10 pmol reverse primer, 1X Superscript III reaction buffer, 0.4 μl of Superscript III enzyme mix and, for primer set 9 only, 10% DMSO in a total volume of 10 μl. For primer set 3, cycling conditions were: reverse transcription at 55°C for 30 min, denaturation at 94°C for 5 min, 38 cycles of denaturation at 94°C for 15 sec, annealing at 60°C for 30 sec and extension at 68°C for 1 min. For primer set 9, cycling conditions were as above except that reverse transcription was carried out at 60°C and the cycle number was increased to 40. *SDHA* (chromosome 5p) and *HPRT1* (Xq26) were used as controls for RT-PCR in samples from which no *VHL* RT-PCR product was obtained. Primers were: *SDHA* forward tgggaaca agagggcatctg and reverse ccaccactgcatcaaattcatg and *HPRT1* forward gacactggcaaaacaatgca and reverse cttcgtggggtccttt tcacc. RT-PCR conditions were as described as for *VHL* primer set 3 except annealing was at 55°C and the cycle number was increased to 40. 4 μl of each RT-PCR product were examined using 1.5% agarose gels in 1X TBE buffer to determine yield and purity.

PCR from trace levels of DNA present in the RNA preparations was carried out in order to confirm the presence of mutations absent from RNA or to compare the relative levels of variants in DNA and RNA. Reactions contained 10 ng of total RNA, 10 pmol forward primer, 10 pmol reverse primer and 5 μl of HotStarTaq master mix (Qiagen) in a total volume of 10 μl. Cycling conditions were: denaturation at 95°C for 15 min, 40 cycles of (denaturation at 95°C for 30 sec, annealing at 60°C for 30 sec and extension at 72°C for 30 sec), final extension at 72°C for 10 min. Primers were as follows: M3537 VHL1_1F cgtc gtgctgcccgtatg; M3563 VHL 1_2F gctgcgctcggtgaactcg; M3538 VHL1_1R accgtgctatcgtccctgct; M3539 VHL2_1F ggctctttaacaacctttgctt; M3540 VHL2_1R tggcaaaaataggctgtcc; M3541 VHL2_2F ccaaactgaattatttgtgccatc; M3542 VHL2_2R tggtctatcctgtactyaccacaacaa; M3543 VHL3_1F gaccctagtctgcc actgagga; M3544 VHL3_1R agagcgacctgacgatgtcc.

### Sequencing

RT-PCR and PCR products were prepared for sequencing by treatment of 2.5 μl of product with 1 μl of ExoSAP-IT (USB, http://www.usbweb.com) and 10 μl sequencing reactions were carried out using 1 μl of prepared PCR product, 1.6 pmol of primer, BigDye ready reaction mix (Applied Biosystems, http://www3.appliedbiosystems.com) version 3.1 diluted 1 in 8 with Half Big Dye reagent (Genetix, http://www.genetix.com/). Cycle sequencing conditions were 25 cycles of 96°C for 10 sec, 50°C for 5 sec and 60°C for 4 min. Unincorporated nucleotides and primers were removed by ethanol precipitation. Sequencing products were resuspended in 20 μl of formamide and run on a 3130 genetic analyser (Applied Biosystems) using POP7 and 36 cm well-to-read capillaries. Data analysis was carried out by visual inspection of electropherograms and using Mutation Surveyor software (SoftGenetics). RT-PCR product 9 and all PCR products were sequenced using the same primers that had been used for RT-PCR/PCR. RT-PCR product 3 was sequenced using forward primer tccacagctaccgaggtcac and reverse primer tctttcagagtatacactgg. These primers bind over the exon 1–2 and exon 2–3 junctions, respectively, and consequently do not bind to the exon 2-lacking product, which co-amplifies in the primer set 3 reaction.

### Methylation analysis

The methylation status of the VHL promoter was examined by methylation-specific PCR (15). Genomic DNA (1 μg) was bisulphite-treated using EZ DNA methylation kit (Zymo Research). Methylated and unmethylated DNA were amplified in separate reactions with primers as previously described (14). CpGenome universal methylated DNA (Millipore) was used as a control. Products were examined by agarose gel electrophoresis.

### Prediction of splice sites and exonic splicing enhancers (ESE)

Splice site prediction by neural network (http://www.fruitfly.org/seq_tools/splice.html) was used to predict the effect of mutations on splice sites. The presence of exonic splicing enhancers was predicted using ESEfinder 3.0 (http://rulai.cshl.edu/cgi-bin/tools/ESE3/esefinder.cgi?process=home) together with possible effects of mutations.

## Results

**Figure 1:**
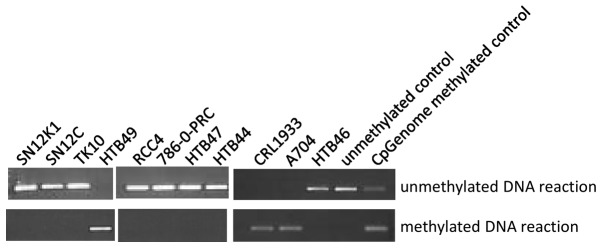
Methylation of VHL promoter in cell line DNA. Eleven cell lines were examined for the presence of methylation at the VHL promoter using methylation-specific PCR. The methylated DNA reaction produces a PCR product of 158 bp from bisulphite-converted methylated DNA. The unmethylated DNA reaction produces a PCR product of 165 bp from bisulphite-converted unmethylated DNA. Neither reaction produces a product from DNA which has not undergone bisulphite conversion (data not shown). The CpGenome methylated DNA control contains both methylated and unmethylated DNA and produces a product with each reaction. All cell lines tested produced a product with only one of the two reactions and hence contain either only methylated or only unmethylated DNA.

The tumour samples were selected to give a distribution of point mutations and premature termination codons (PTCs) throughout the 3 coding exons of *VHL* (Table I) with samples from 56 patients containing 52 unique VHL mutations or polymorphisms (Table IA–C) together with a further 28 tumours (24 ccRCC and 4 other subtypes) in which no variant had been identified. In the tumour samples, based on the predicted change to the primary amino acid sequence, 24 of the mutations were missense, 11 resulted in frameshifted amino acid sequence followed by a premature truncation codon (PTC), 9 resulted in immediate truncation at the site of the mutation and 2 were frameshifts which extended the reading frame beyond the normal stop codon into the 3′UTR. Tumours with intronic variants (Table IC) included three tumours with intronic substitutions affecting invariant residues at splice sites, one tumour with a deletion which removed nucleotides spanning the exonintron junction (R1T), and one tumour with an intronic variant of unknown function (R281T). An intronic polymorphism, c.463+43 A>G (rs34661876) was present in 5 tumours. This polymorphism and two of the missense variants (p.D9N and p.P25L (rs35460768)) were germline in origin, all of the other variants were somatic (6,14). p.P25L is not associated with VHL disease (3). p.D9N has not, to our knowledge, been reported previously, but affects a non-conserved residue at the extreme N-terminus and is unlikely to be associated with VHL disease. Additionally 11 RCC cell lines were analysed. Truncating mutations were identified in three cell lines (Table IA), missense mutations in a further three (Table IB), while five had no detectable mutation (Table ID). All mutations detected were homozygous. Although mutation status of many of these cell lines has previously been reported, mutation nomenclature may have used alternative nucleotide numbering systems, so for consistency we have included these results in Table I. In the cell lines CRL1933, A704 and HTB49 the VHL promoter was methylated (Fig. 1) and no *VHL* RT-PCR product was found in these 3 cell lines only although control reactions confirmed the presence of amplifiable RNA in these samples (data not shown). CRL1933 and A704 were also homozygously mutated, showing that mutation and methylation can occur on the same allele.

**Figure 2:**
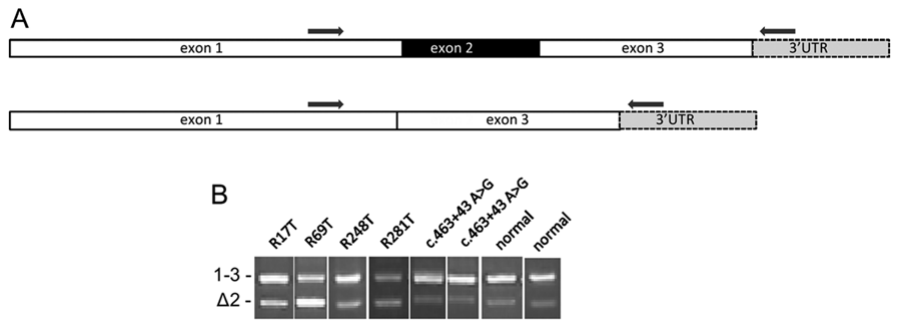
Agarose gel images of RT-PCR products. (A) Schematic showing the two transcripts produced from the VHL gene. Amplification using primer set 3, in which the primers are located in exons 1 and 3, produces two products. The larger product, 1–3, (442 bp) is produced from full length VHL mRNA. The smaller product, Δ2, (319 bp) lacks exon 2. (B) Agarose gel images of RT-PCR products showing 1–3 and Δ2 products. In normal kidney tissue, the Δ2 product is much weaker than the full length product. The same pattern was observed in the majority of the tumour samples (data not shown). We observed four tumours in which the relative intensity of the bands was notably different. Tumour R69T had a clearly brighter lower band; in tumour R281T both bands were of similar intensity and in tumours R17T and R248T, although the full length band was stronger, there was less difference in intensity between the two bands than in control samples. Also shown are two tumours which contain the common polymorphism c.463+43 A>G. As this image is compiled from several individual gels, the absolute distance separating the two bands may differ but in every case the mobility relative to molecular weight markers and to controls was the same.

*VHL* produces two mRNA species (2); the full length product consisting of exons 1–3 and a shorter product lacking exon 2 (Fig. 2A). No aberrantly sized bands indicating an effect on pre-mRNA splicing were seen in any sample other than R222T which has 26 bp duplication (data not shown); smaller insertion or deletion mutations present in other samples were below the resolving power of the system. An increased intensity of the lower band when compared to the upper band was seen in four tumours (Fig. 2B). R17T and R69T both have exonic mutations, c.343C>A and c.341G>C respectively, located close to the exon 2 splice acceptor site. R281T contains two variants, c.463+43A>G and c.341-11T>A. None of the tumours with c.463+43A>G alone showed any change to the product ratios, suggesting that the rare somatic variant c.341-11T>A could be responsible for the change. One other tumour, R248T, in which we had not previously detected a variant, showed a more subtle change to the band ratio.

The *VHL* RT-PCR products were sequenced in order to confirm use of the correct splice sites and to determine whether exonic mutations were present in the message. Splice site usage was exclusively at the wild-type sites, no activation of local cryptic splice sites was observed. Mutant RNA containing the expected exonic variant was detected in all 13 tumours containing frameshift variants, 9/10 tumours containing nonsense variants and 21/24 tumours containing missense variants (Table IA and B). Mutant RNA was also detected in each of the four cell lines which had mutations and expressed VHL RNA. There were four tumours in which we were unable to detect the previously identified mutation. One of these tumours had a nonsense variant, c.400G>T/p.E134^*^; the other three tumours had the missense variants c.340G>C/p.G114R, c.341G>C/p.G114D and c.461C>T/p.P154L. Previous work showed methylation of the VHL promoter in the tumour with c.340G>C but not in the other three tumours (6,14).

The tumour R1T has a 10 bp deletion, c.332_340+1del10, which removes the final 9 nucleotides of exon 1 and the first nucleotide of the splice donor site in intron 1. Deletion of a splice site would be expected to prevent splicing, and splice site prediction software indeed predicts that the splice site is completely destroyed (Table II). Sequencing of the RT-PCR product from R1T, as expected, showed only wild-type sequence, consistent with the mutant being unable to form a mature *VHL* mRNA.

**Figure 3:**
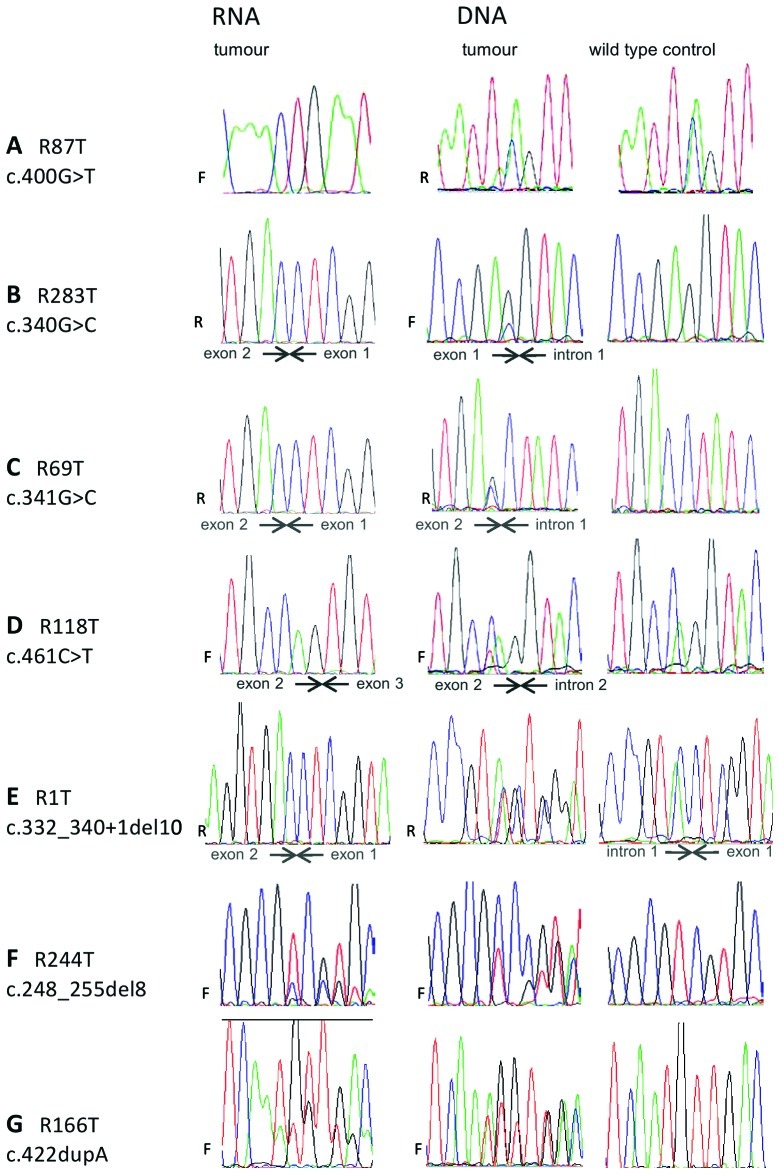
Mutations absent from VHL RT-PCR products. The left column shows sequencing traces of VHL RT-PCR products from seven tumours. The centre column shows sequencing traces of VHL PCR products amplified from the co-purifying DNA and shows the presence of the expected variant in each case compared to a wild-type control sample (right column). Exon-exon and intron-exon boundaries are indicated. Forward or reverse orientation of the trace is indicated and may differ between RNA and DNA sources. In panels A–D, each mutation is located in an exon and its absence from RNA was unexpected. In each case, the mutation is clearly present in DNA. In panel E, the mutation deletes the exon 1 donor splice site and mature mutant message was not expected. Again, the mutation is clearly present in tumour DNA. Panels F and G show data from two tumours in which a substantial difference in mutation level was observed between RNA and DNA. In each case, the mutation is present as the minor fraction in RNA but the major fraction in DNA.

Sequencing of the RT-PCR products from the 28 tumours in which no mutation had previously been identified revealed the presence of mutations in two tumours, c.350G>A and c.315_330del16 (Table IA). In the five mutant samples in which no mutation was present in RNA and the two samples in which a mutation was newly identified in the RNA, co-purifying DNA was examined. In R1T, from which the absence of the mutation from RNA was not unexpected, as well as each of the four samples where the expected mutation was not detected in RNA, the mutation was clearly present in DNA, confirming that the sample contained sufficient tumour material for the mutation to be detectable (Fig. 3A–E). In the two samples in which mutations were newly detected, the mutations were clearly present in DNA (data not shown) and our failure to detect these in our previous study presumably reflects the heterogeneity of the tissue samples.

Nonsense-mediated decay may reduce the level of mutant RNA in tumours containing premature termination codons (PTCs). We compared the level of mutation in RNA and DNA in samples containing PTCs and expressing mutant RNA except those where the mutation was located 5′ of nucleotide c.208, as no satisfactory PCR product could be obtained from these samples. In each case, the expected mutation was present in co-purifying DNA. However, the level of mutation observed in the DNA sequencing traces from R117T, R166T, R244T, R246T, R248T and R255T was markedly different from the level observed in RNA sequencing traces (Fig. 3 F–G, Table I).

Splice site prediction software (16) was used to examine whether there was a change to the predicted strength of the splice sites of variants located in introns, within the few nucleotides adjacent to introns or in samples which had altered ratios of RT-PCR products (Table II). For the four variants which affected absolutely conserved residues at splice sites, the software predicted that the variant eliminated the existing splice site. However, none of these samples had any evidence for exon skipping as determined by altered RT-PCR product ratios. Five variants either produced no change or increased the strength of the prediction. These were the common variant c.463+43A>G, the substitutions c.343C>A, c.343C>G and c.350G>A which are located 3–10 nucleotides into exon 2 and c.464_474 del 11 which removes the first 11 nucleotides of exon 3. Of these, c.343C>A and c.350G>A had altered band ratios by RT-PCR suggestive of exon skipping whereas the others had a normal appearance. Four variants had reduced scores and of these c.341G>C and c.341-11T>A had a RT-PCR band ratio suggestive of exon skipping whereas c.340G>C and c.461C>T had a normal appearance. The possibility of c.341-11T>A introducing a cryptic splice site was considered but although the donor splice site consensus sequence YAG is introduced 9 nucleotides 5′ of the true exon 2 donor site, the software did not predict the creation of a site and no RT-PCR products using this cryptic site were observed. Finally, the variant c.400G>T, in which mutant RNA is absent was examined, and no creation of a cryptic splice site was predicted (data not shown).

Exonic splicing enhancers (ESEs) are cis-acting signals located within exons which act as binding sites for proteins essential for splicing. Using ESE-finder software (17) to examine the predicted effects of the mutations c.340G>C, c.341G>C, c.343C>T, c.350G>A, c.400G>T and c.461C>T which have experimental evidence to suggest that they perturb RNA processing, only c.341G>C and c.461C>T showed any changes in predicted ESEs: an SF2/ASF binding site was created in c.341G>C and an SF2/ASF binding site was replaced with a SC35 binding site in c.461G>C.

## Discussion

Examination of *VHL* mRNA in RCC clearly complements genomic analyses illustrating the unpredictability of some findings and the potential for multiple truncated forms of VHL protein based on the presence of transcript. Four tumours showed an increase in the Δ2 product relative to the full length RT-PCR product. R17T and R69T both have exonic mutations, c.343C>A and c.341G>C respectively, located close to the exon 2 splice acceptor site (discussed further below). In R281T, c.341-11T>A is the only rare variant we have detected. Predictions using this sequence change (Table II) suggest a small decrease in the strength of the exon 2 splice acceptor, which may be able to account for the exon skipping observed. This mutation is within the polypyrimidine tract, a loosely defined sequence which is involved in 3′ splice site definition (18). Rare reports of polypyrimidine tract mutations exist (19). R248T contains the exonic variant c.350G>A. No changes to splice site strength (Table II) or to ESE sequences were predicted and the cause of exon skipping is unclear. PCR bias towards shorter products could contribute toward a bias to under-reporting of use of distant cryptic sites and intron retention. However, exon skipping is the most common consequence of mutation-related aberrant splicing, followed by use of local cryptic sites, with intron retention accounting for only a minority of cases (20,21). Other reported examples of aberrant splicing of *VHL* also describe mutations which cause skipping of exon 2 (2,22). As germline deletion of exon 2 alone is sufficient to cause VHL disease (23), Δ2 is unlikely to retain *VHL* function. Four tumours (R1T, R86T, R139T, R167T) have mutations which affect at least one of the absolutely conserved nucleotides within consensus splice sites (24) and all are predicted to remove the affected splice site (Table II). Interestingly, none of the *VHL* RNA from of these tumours showed an increase in the Δ2 isoform. The exon skipping mutations reported previously (2,22) affect either conserved but not invariant intronic residues or exonic residues, as do the mutations that we have observed which cause exon skipping.

Coding exon sequences describe the primary amino acid sequence of the protein but the same nucleotides may also contain information required for the correct processing of gene transcripts (reviewed in ref. 25). Mutations which appear to be silent, missense, nonsense or frameshift in their effects on the amino acid sequence, may instead act by disrupting RNA processing by a variety of mechanisms including creation of cryptic splice sites (26,27), disruption of ESE sequences (28,29) and disruption of the exonic portion of consensus splice sites (30,31). We observed three exonic substitutions (c.340G>C, c.341G>C and c.461C>T) which encode missense changes to the primary amino acid sequence and in which mutant RNA was not detectable (Fig. 3). These mutations are located, respectively, in the final nucleotide of exon 1, the first nucleotide of exon 2 and at the −3 position of exon 2. Two further mutations included in this study were located at the +3 position of exon 2: c.243C>A and c.243C>G, of which c.243C>A but not c.243C>G showed evidence for exon skipping. The three tumours which had no detectable mutant RNA all had mutations which reduced the predicted strength of the splice site (Table II). Exonic nucleotides immediately adjacent to the consensus splice site sequences have non-random nucleotide sequences, with the 3′-most nucleotide of the exon having the greatest sequence constraint (24,32). Disease-causing mutations are reported in most of these positions, most frequently at the 3′-most nucleotide of the exon (21).

As c.340G>C, c.341G>C and c.461C>T do not produce mutant mRNA, they must therefore function solely as splicing mutations. c.243C>A appears to be associated with some degree of exon skipping but is still detectable in *VHL* mRNA and may produce its effects through multiple mechanisms including splicing and missense change to pVHL whereas c.243C>G does not appear to manifest any deleterious consequences at the RNA level.

Introduction of a PTC into a gene product accounts for a substantial proportion of the mutations underlying both inherited genetic disease and many forms of cancer (33). Of familial VHL mutations, 13% are frameshift and 11% are nonsense (3) while in sporadic RCC we have found 51% of detected mutations to be frameshift and 11% nonsense (6). Nonsense-mediated decay (NMD) (reviewed in refs. 34,35) degrades mRNAs which contain PTCs, protecting cells from potentially deleterious consequences of truncated proteins. Numerous studies report severe reduction in the level of mRNAs containing nonsense codons (36–38). However, not all PTCs elicit NMD and the level of reduction can be highly variable (39,40). Location of the PTC in the transcript can influence susceptibility to NMD (39,40) with PTCs in the 3′ terminal exon or within 50–54 nucleotides of the final exon-exon junction not triggering NMD (41). In the case of *VHL*, only those mutations which introduce PTCs into exon 1 or into the 5′ end of exon 2 (before codons 137–138) could trigger NMD. Because *VHL* contains fairly long ORFs in both alternative reading frames, it is possible for the PTC associated with a mutation to be located some considerable distance downstream of the mutation. Two of the tumours in this study (R189T and R231T) have sufficiently long ARFs that the PTC is located after the 50–54 nt boundary, although the mutation itself is located well before the boundary.

Previously, two cell lines with frameshift mutations which terminated after the 50–54 nt boundary were shown not to be susceptible to NMD (42). Of four other mutations examined (43), two of which were of the intronless *Drosophila Vhl* gene, the data showed that in both humans and *Drosophila*, the presence of a truncating mutation does not necessarily trigger NMD and that the site of the PTC within the transcript was likely to be the factor which determined whether NMD occurred (43). In 22 of 23 RCC tumours containing PTC mutations, mutant mRNA was readily detectable (Table IA). In one tumour, which contained the nonsense-variant p.E134^*^, mutant mRNA was undetectable (Table IA) and as the PTC associated with this mutation is located just 5′ of the 50–54 nt boundary, NMD is a plausible explanation. In a further 6 tumours out of 15 in which the relative abundance of mutant RNA and DNA was estimated, there was evidence to support a detectable reduction in the amount of mutant RNA. Five of the seven tumours showing evidence for loss of mutant mRNA had PTCs which were located more than 50–54 nucleotides upstream of the final exon-exon boundary. The two tumours (R189T and R231T) in which a mutation located prior to the 50–54 nt boundary introduces a PTC after the boundary did not show any evidence for reduction of mutant RNA compared to mutant DNA. The cell lines 786-0-PRC, HTB47 and HTB44 contain truncating mutations, all of which result in a PTC located 3′ of the 50–54 nt boundary and all expressed mutant *VHL* RNA. Our study greatly expands the number of truncating *VHL* mutations which have been examined at the RNA level. Our data suggest that in the majority of RCC tumours in which *VHL* has a truncating mutation, even if the PTC is located before the 50–54 nt boundary, mutant mRNA is present, albeit at a reduced level in some tumours. There is therefore the potential for the production of truncated VHL protein in these RCCs which may exhibit partial function. This could ideally be checked by western blotting providing antibodies but would ideally require a range of antibodies to cover unaffected epitopes and additionally our studies find that currently available antibodies are only able to detect VHL protein when grossly overexpressed in transfectant cell lines and not at endogenous levels.

In our previous study with 74.6% of RCC cases having a detectable *VHL* mutation and 31.3% methylation of the *VHL* promoter (6), 32 tumours had both mutation and methylation and of these, 30 also had LOH. Although other studies have observed methylation and mutation to be mutually exclusive (5), it is possible, especially where LOH has also occurred, that mutation and methylation have occurred on the same allele, although tumour heterogeneity may also explain the finding. Our finding that CRL1933 and A704 contain only mutant *VHL* and have only methylated DNA show mutation and methylation are not mutually exclusive in these cell lines. The link between *VHL* promoter hypermethylation and silencing of the gene is well recognised (44) and all cell lines showing promoter methylation had complete absence of *VHL* RNA (Fig. 1). Seven of the 23 tumours containing truncating mutations (30.4%) and 8 of 24 tumours with missense mutations (33.3%) also had methylation providing an alternative explanation for the lack of or reduced level of detectable mutant RNA. However, 3 of the four tumours in which the exonic mutation was not detected in RNA were not methylated.

Eight of the 9 tumours with intronic variants also had methylation (88.9%) which differs markedly not only to the other two categories and but also to the average for unselected tumours. It is, perhaps, unsurprising that there should be a high level of methylation of tumours with the polymorphism c.463+43A>G. It is more surprising that all of the tumours with intronic splicing mutations should also have methylation although possibly a consequence of the small numbers. As germline mutations of *VHL* splice sites are sufficient to cause VHL disease (45), there is no reason to suppose that splicing mutations are less inactivating than other types of mutation.

Deep intronic mutations activating the splicing of cryptic exons (46,47) are more readily detected using RNA than genomic DNA, as conventional mutation scanning of introns is expensive and time-consuming. To our knowledge, deep intronic mutations have not been reported in VHL disease (3) or in cancer (http://www.sanger.ac.uk/genetics/CGP/cosmic/). In the 28 tumours without VHL mutation in our previous study, mutations were identified in two using RNA. However, as they were subsequently confirmed in DNA, we speculate that they were not detected previously due to inadequate tumour cell numbers. Had we used RNA-level mutation screening alone, we would have been unable to detect the presence of the four exonic mutations which prevented the production of mature, stable VHL mRNA or of the four mutations affecting invariant consensus splice site sequences that did not show an altered mRNA isoform ratio. Clearly RNA screening complements DNA mutation screening. Our data show that the biological consequences of *VHL* mutations are not necessarily predictable from the sequence change of the mutation and that for the majority of VHL truncating mutations, the potential for the generation of mutant protein exists. Challenges due to the low level of endogenous VHL protein and the paucity of good VHL antibodies recognising the various domains currently limit study at the protein level but clearly expression studies coupled with functional studies may shed further light on the consequences of the genetic and epigenetic changes in VHL and their potential clinical significance.

## Figures and Tables

**Table I:** Mutation, methylation and RNA expression status in renal tumour tissue and cell lines.

A, Tumours and cell lines with nonsense or frameshift mutations.									
Sample ID	Mutation (cDNA)	Mutation (amino acid)	VHL RNA present	Mutant present in VHL RNA	Estimated % tumour cells in sample	Methylation at VHL promoter detected [tumour data from (6,14)]	Estimated relative abundance of mutation in DNA and RNA	Site of predicted termination (codon no.)	Length of frameshift (no. aa)
R215T	c.171delG	p.R58fs	+	+	60	−	Not tested	66	8
R197T	c.183delC	p.V62fs	+	+	20–40	+	Not tested	66	4
R35T	c.194C>A	p.S65^*^	+	+	60	−	Not tested	65	n/a
R271T	c.194C>A	p.S65^*^	+	+	75	+	Not tested	65	n/a
R19T	c.203C>A	p.S68^*^	+	+	25	−	Not tested	68	n/a
R138T	c.208G>T	p.E70^*^	+	+	45–80	−	Not tested	70	n/a
R189T	c.226_235del10	p.F76fs	+	+	70	−	DNA≈RNA	155	79
R244T	c.248_255del8	p.V83fs	+	+	80	−	DNA>RNA	128	45
R236T	c.274_278del5	p.D92fs	+	+	75	−	DNA>RNA	129	37
R231T^a^	c.315_330del16	p.G106fs	+	+	75	+	DNA≈RNA	153	47
R243T	c.337C>T	p.R113^*^	+	+	50	−	Not tested	113	n/a
R248T^a^	c.350G>A	p.W117^*^	+	+	60–70	+	DNA>RNA	117	n/a
R255T	c.360_367del8	p.A122fs	+	+	40	+	DNA>RNA	128	6
R87T	c.400G>T	p.E134^*^	+	−	60	−	n/a	134	n/a
R224T	c.404dupT	p.L135fs	+	+	70	+	DNA≈RNA	143	8
R166T	c.422dupA	p.N141fs	+	+	75	−	DNA>RNA	143	2
R181T	c.443_444delTT	p.F148fs	+	+	30–50	−	RNA>DNA	172	24
R117T	c.464_474del11	p.V155fs	+	+	60	−	DNA>RNA	169	14
R182T	c.467dupA	p.Y156^*^	+	+	75	−	RNA>DNA	156	n/a
R222T	c.473_498dup26	p.R167^*^	+	+	75	−	RNA>DNA	167	n/a
R177T	c.477delA	p.E160fs	+	+	80	+	DNA≈RNA	169	9
R134T	c.481C>T	p.R161^*^	+	+	10–40	−	DNA≈RNA	161	n/a
R164T	c.525C>A	p.Y175^*^	+	+	50	−	DNA≈RNA	175	n/a
R202T	c.563dupT	p.E189fs	+	+	60–70	−	Not tested	3′UTR	66
R252T	c.620_635dup16	p.D213fs	+	+	30	−	Not tested	3′UTR	47
Cell lines									
786-0^b,c^	c.311delG	p.G104fs	+	+	n/a	−	n/a	158	54
HTB47^b,d^	c.529A>T	p.R177^*^	+	+	n/a	−	n/a	177	n/a
HTB44^b,e^	c.426_429del4	p.G144fs	+	+	n/a	−	n/a	158	13

B, Tumours and cell lines with missense mutations.						
Sample ID	Mutation (cDNA)	Mutation (amino acid)	VHL RNA present	Mutant present in VHL RNA	Estimated % tumour cells in sample	Methylation at VHL promoter detected [tumour data from (6,14)]
R185T^k^	c.25G>A	p.D9N	+	+	80	−
R187T	c.74C>T	p.P25L	+	+	40–60	+
R124T	c.232A>G	p.N78D	+	+	70–85	−
R157T	c.256C>G	p.P86A	+	+	80	−
R247T	c.262T>A	p.W88R	+	+	80	+
R261T	c.269A>T	p.N90I	+	+	80	−
R292T	c.302T>C	p.L101P	+	+	75	−
R160T	c.320_321delinsCA	p.R107P	+	+	85–90	−
R283T	c.340G>C	p.G114R	+	−	40–60	+
R69T	c.341G>C	p.G114D	+	−	75	−
R10T	c.343C>G	p.H115D	+	+	90	+
R17T	c.343C>A	p.H115N	+	+	50	−
R273T	c.349T>C	p.W117R	+	+	80	−
R183T	c.361G>T	p.D121Y	+	+	65–70	+
R267T	c.388G>C	p.V130L	+	+	95	+
R142T	c.406T>G	p.F136V	+	+	65–90	−
R295T	c.413C>G	p.P138R	+	+	80	−
R294T	c.446C>A	p.A149D	+	+	70	−
R123T	c.452T>A	p.I151N	+	+	75 (s1 necrotic)	−
R118T	c.461C>T	p.P154L	+	−	40	−
R151T	c.473T>A	p.L158Q	+	+	60–75	−
R279T	c.485G>A	p.C162Y	+	+	75	+
R218T	c.497T>G	p.V166G	+	+	70	+
R200T	c.551T>C	p.L184P	+	+	60	+
Cell lines						
CRL1933^b,f^	c.539T>A	p.I180N	−	n/a	n/a	+
RCC4^b,g^	c.194C>G	p.S65W	+	+	n/a	−
A704^b,h^	c.539T>A	p.I180N	−	n/a	n/a	+

C, Tumours with intronic mutations.						
Sample ID	Mutation (cDNA)	Mutation (amino acid)	VHL RNA present	Mutant present in VHL RNA	Estimated % tumour cells in sample	Methylation at VHL promoter detected [tumour data from (6,14)]
R1T	c.332_340+1del10	n/a	+	−	65	+
R86T	c.340+1G>T	n/a	+	−	90	+
R167T	c.340+2delT	n/a	+	−	75	+
R139T	c.341-1G>A	n/a	+	−	45	+
R281T	c.[ 341-11T>A (;)463+43A>G]	n/a	+	−	50–60	+
R179T	c.463+43A>G	n/a	+	−	80	+
R230T	c.463+43A>G	n/a	+	−	75	+
R233T	c.463+43A>G	n/a	+	−	60	+
R276T	c.463+43A>G	n/a	+	−	80	−

D, Tumours and cell lines without mutations.						
Sample ID	Mutation (cDNA)	Mutation (amino acid)	VHL RNA present	Mutant present in VHL RNA	Estimated % tumour cells in sample	Methylation at VHL promoter detected [tumour data from (6,14)]
Tumours						
R108T^k^	No mutation detected	n/a	+	n/a	95	−
R128T	No mutation detected	n/a	+	n/a	80	−
R140T	No mutation detected	n/a	+	n/a	35–75	−
R143T	No mutation detected	n/a	+	n/a	10	−
R150T	No mutation detected	n/a	+	n/a	40	−
R153T^k^	No mutation detected	n/a	+	n/a	85	−
R158T^k^	No mutation detected	n/a	+	n/a	70	−
R163T	No mutation detected	n/a	+	n/a	80–90	−
R165T	No mutation detected	n/a	+	n/a	80–85	−
R195T	No mutation detected	n/a	+	n/a	95	−
R201T	No mutation detected	n/a	+	n/a	20–30	−
R203T	No mutation detected	n/a	+	n/a	90	−
R207T	No mutation detected	n/a	+	n/a	n/d	−
Tumours						
R208T	No mutation detected	n/a	+	n/a	n/d	−
R217T	No mutation detected	n/a	+	n/a	80	−
R220T	No mutation detected	n/a	+	n/a	70	−
R225T^k^	No mutation detected	n/a	+	n/a	0	−
R226T	No mutation detected	n/a	+	n/a	75–85	−
R228T	No mutation detected	n/a	+	n/a	65–75	−
R230T	No mutation detected	n/a	+	n/a	80–95	+
R242T	No mutation detected	n/a	+	n/a	30–50	+
R253T	No mutation detected	n/a	+	n/a	80–90	−
R256T	No mutation detected	n/a	+	n/a	60	+
R280T	No mutation detected	n/a	+	n/a	70–75	−
R282T	No mutation detected	n/a	+	n/a	90	+
R290T	No mutation detected	n/a	+	n/a	90	+
Cell lines						
HTB46^b,i^	No mutation detected	n/a	+	n/a	n/a	−
SN12C^b,j^	No mutation detected	n/a	+	n/a	n/a	−
TK10^b,j^	No mutation detected	n/a	+	n/a	n/a	−
HTB49^b^	No mutation detected	n/a	−	n/a	n/a	+
U031^b,j^	No mutation detected	n/a	+	n/a	n/a	n/d

Mutations are described using guidelines at http://www.hgvs.org/mutnomen/ and the reference sequence used corresponds to NM_000551 or ENST00000256474, in each case with A of ATGi as nucleotide 1.

a Mutation newly identified during RNA screening.

b Cell line. All mutations detected in cell lines were homozygous.

c Mutation previously reported (2,48).

d HTB47 is also known as Caki-2; mutation previously reported (48).

e HTB44 is also known as A498; mutation previously reported (1,2,48).

f CRL1933 is also known as 769P; mutation previously reported (48).

g Mutation previously reported (49).

h Mutation previously reported (48).

i HTB46 is also known as Caki-1; previously screened (50).

j Previously screened (50).

k Non-clear cell RCC.

**Table II:** Predicted effects on splice site strength using http://www.fruitfly.org/seq_tools/splice.html.

Splice site	Tumour	Variant	Predicted strength
Exon 1 donor		Wild-type	0.99
	R1T	c.332_340+1 del 10	No site predicted
	R283T	c.340 G>C	0.75
	R86T	c.340+1 G>T	No site predicted
	R167T	c.340+2 del T	No site predicted
Exon 2 acceptor		Wild-type	0.97
	R281T	c.341-11 T>A	0.88
	R139T	c.341-1 G>A	No site predicted
	R69T	c.341 G>C	0.85
	R17T	c.343 C>A	0.97
	R10T	c.343 C>G	0.99
	R248T	c.350 G>A	0.97
Exon 2 donor		Wild-type	0.82
	R118T	c.461 C>T	0.46
	R179T, R230T, R233T,	c.463+43 A>G	0.82
	R276T, R281T		
Exon 3 acceptor		Wild-type	0.84
	R117T	c.464_474 del11	0.96
